# Prestimulus α/β power in temporal-order judgments: individuals differ in direction of modulation but show consistency over auditory and visual tasks

**DOI:** 10.3389/fncom.2023.1145267

**Published:** 2023-05-25

**Authors:** Lars T. Boenke, Abdelhafid Zeghbib, Myra Spiliopoulou, David Alais, Frank W. Ohl

**Affiliations:** ^1^Leibniz Institute for Neurobiology (LIN), Magdeburg, Germany; ^2^School of Psychology, University of Sydney, Sydney, NSW, Australia; ^3^Department of Automatic Control and Systems Engineering (ACSE), University of Sheffield, Sheffield, United Kingdom; ^4^National Institute for Physiological Sciences (NIPS), Okazaki, Japan; ^5^Research Lab Knowledge Management and Discovery, Faculty of Computer Science, Otto-von-Guericke University, Magdeburg, Germany; ^6^Faculty of Science, Otto-von-Guericke University, Magdeburg, Germany; ^7^Center for Behavioral Brain Sciences (CBBS), Magdeburg, Germany

**Keywords:** active inhibition, complex systems, consistency, gating, implicit assumptions, inter-individual variability, replicability, reproducibility

## Abstract

The processing of incoming sensory information can be differentially affected by varying levels of α-power in the electroencephalogram (EEG). A prominent hypothesis is that relatively low prestimulus α-power is associated with improved perceptual performance. However, there are studies in the literature that do not fit easily into this picture, and the reasons for this are poorly understood and rarely discussed. To evaluate the robustness of previous findings and to better understand the overall mixed results, we used a spatial TOJ task in which we presented auditory and visual stimulus pairs in random order while recording EEG. For veridical and non-veridical TOJs, we calculated the power spectral density (PSD) for 3 frequencies (5 Hz steps: 10, 15, and 20 Hz). We found on the group level: (1) Veridical auditory TOJs, relative to non-veridical, were associated with higher β-band (20 Hz) power over central electrodes. (2) Veridical visual TOJs showed higher β-band (10, 15 Hz) power over parieto-occipital electrodes (3) Electrode site interacted with TOJ condition in the β-band: For auditory TOJs, PSD over central electrodes was higher for veridical than non-veridical and over parieto-occipital electrodes was lower for veridical than non-veridical trials, while the latter pattern was reversed for visual TOJs. While our group-level result showed a clear direction of prestimulus modulation, the individual-level modulation pattern was variable and included activations opposite to the group mean. Interestingly, our results at the individual-level mirror the situation in the literature, where reports of group-level prestimulus modulation were found in either direction. Because the direction of individual activation of electrodes over auditory brain regions and parieto-occipital electrodes was always negatively correlated in the respective TOJ conditions, this activation opposite to the group mean cannot be easily dismissed as noise. The consistency of the individual-level data cautions against premature generalization of group-effects and suggests different strategies that participants initially adopted and then consistently followed. We discuss our results in light of probabilistic information processing and complex system properties, and suggest that a general description of brain activity must account for variability in modulation directions at both the group and individual levels.

## Significance statement

Despite intense debates and important contributions, the role of variability observed at all levels of biological organization (including replication) is only now gaining momentum in some subfields of neuroscience. For example, a common assumption is that a reduction in prestimulus power, particularly in the 10–20 Hz frequency range, is associated with improved perception. Many studies agree, but recent discrimination studies in particular often show a null-result. Worse, in an auditory temporal-order judgment (TOJ) task, even the opposite has been reported. Here, we had participants perform auditory and visual TOJ presented in unpredictable order. For both conditions, we found a group-level association of an increase in prestimulus power and better (spatial-temporal) discrimination (replicating the previous auditory TOJ study). However, at the individual level, the results reflect the situation in the literature: the same effect (veridical TOJs, i.e., when the temporal order of signals was reported according to the presented physical order of signals) was associated with an increase in prestimulus power in some and with decreased power in others. Importantly, individuals were consistent across conditions, indicating that they consistently maintained a strategy once adopted. This is an important contribution to how to better understand some of the seemingly contradictory data in the literature. Some of the observed variability is not “noise” but a characteristic of complex systems that can realize certain effects through a multiplicity of pathways.

## 1. Introduction

At least since Berger’s (1929) first published measurement of electrophysiological activity from the human scalp, there have been many efforts to understand the relationship between neuronal oscillations and brain function and cognition. A large volume of research has pushed the boundaries of our knowledge. However, there are also recent studies, whose results question with previous assumptions (see e.g., Iemi et al., 2017; Keitel et al., 2022 for a review). A prominent example of controversy among study results concerns the direction of modulation of α-power before the stimulus, which is also the focus of our work. In this work, we report on results that explain this disagreement by shedding light on the relationship between prestimulus power modulation and inter-individual differences. Before proceeding with the presentation of our study, we provide below a brief overview of today’s prevailing assumptions, controversial results, and recent developments.

Oscillations in neural activity are believed to be a signature of information integration across distributed networks (Fries, 2005; Hipp et al., 2012), with different oscillation frequencies linked to specific cognitive operations. For example, selective attention is systematically linked to oscillations in the theta (∼4–8 Hz) to gamma (>30 Hz) range [reviewed in Womelsdorf and Fries (2007)]. Within this range, the α-band (∼8–13 Hz) is most dominant. Due to its dominance, a significant increase in α-power with eyes closed compared to eyes open was described already in the early days of EEG research, leading to the so-called idling hypothesis [reviewed in Pfurtscheller et al. (1996)]. In a series of further studies, it then became clear that the α-band modulation reflects more than just idling at rest (e.g., Kelly et al., 2006). Nowadays, several studies suggest that α-power is bidirectionally modulated; both increased and decreased α-power have been reported as a function of task demands (cf. Klimesch, 2012). For example, (actively) suppressed prestimulus α-oscillations have been reported to be associated with enhanced recognition of near-threshold visual stimuli (e.g., Ergenoglu et al., 2004), improved discrimination performance (e.g., van Dijk et al., 2008) and that lateralized cues increase ipsilateral α-power and decrease contralateral α-power (e.g., Rihs et al., 2007; Wyart and Tallon-Baudry, 2008). These findings lead to the now widespread view that the modulation of α-power prior to a stimulus is related to (selective) attentional processes that serve as an active gating mechanism (e.g., Jensen and Mazaheri, 2010; Foxe and Snyder, 2011; Klimesch, 2012). In this notion, upregulation and downregulation go hand in hand in task-irrelevant and task-relevant networks, respectively. Whereas increased prestimulus α-power decouples task-irrelevant neural structures from task-relevant structures, thus minimizing distractor processing, decreased α-power prior to the stimulus is viewed as increased excitability of neural networks (review: Samaha et al., 2020), reflecting a greater readiness to process task-relevant information.

Active inhibition as gating is thought to extend beyond selective processing within a single modality. This idea has been pursued, for example, in studies attempting to modulate prestimulus α-power in an intermodal selective attention task by directing attention to either the auditory or visual modality (Foxe et al., 1998; Fu et al., 2001).

Although the active inhibition hypothesis (AIH) is elegant and a large chunk of available data can be explained by it, the empirical evidence is not always as clear-cut as one might like to wish: for example, in the study by Foxe et al. (1998), parieto-occipital electrodes showed increased α-power when the auditory modality was cued, consistent with the concept of active inhibition, but there was no upregulation across central (reflecting auditory processing) electrodes when the visual modality was cued (see also van Diepen et al., 2015). That is, the expected cadence of upregulation in the irrelevant modality and downregulation in the relevant modality predicted by the hypothesis in its strong form was not supported by the data.

Although anatomical factors may complicate the recording of α-power in auditory cortex (Bastiaansen and Knösche, 2000; for a review of the debate: Weisz et al., 2011; for further potential differences between auditory and visual modality in this context see Zoefel and VanRullen, 2017), this may not be the whole story: the opposite pattern, namely a measurable α-modulation in auditory but correspondingly not in visual cortex, was also reported. Grabot et al. (2017) had participants judge the order of audiovisual stimulus pairs and examined the modulation of α-power prior to the stimulus using magnetic encephalography. Based on the idea of prior entry (Titchener, 1908; Spence and Parise, 2010) that a tendency to report stimuli within a given modality as perceived earlier is attributable to increased attention to that modality, the authors expected a relative increase in α-activity in auditory cortex compared to visual cortex prior to stimulus onset when participants tend to report the visual stimulus earlier (decoupling the less attended auditory modality from the attended visual modality). Accordingly, they expected relatively higher α-activity in prestimulus visual cortex compared to auditory cortex when participants are inclined to report the auditory stimulus as perceived earlier (decoupling the less attended visual modality from the attended auditory modality). However, this was not the case. Instead, the authors found only relatively lower activation of prestimulus α-activity in auditory cortex associated with veridical audiovisual judgments of temporal order (TOJs). Importantly, they found no corresponding upregulation of α-activity in visual cortex before the period of stimulus onset, let alone any substantial and task-related modulation of α-activity in the prestimulus period outside auditory cortex.

Further complicating matters are studies that report the opposite of the expected direction of modulation [Womelsdorf and Fries, 2007; see the discussion in Haegens et al. (2011)]. For example, Mo et al. (2011) reported that α-band oscillations can differ in sensory cortices and inferotemporal (IT) cortex, the latter of which did not show the expected pattern of attentional suppression in an intermodal audiovisual attention task. Whereas in the visual sensory cortices, α-band power was negatively correlated with reaction times during the detection of auditory stimuli, this pattern was reversed in IT. Other studies reported that α-band modulation was different in the different laminar layers (Buffalo et al., 2011). When Babiloni et al. (2006) asked their participants in a visual go/no-go paradigm whether they had perceived a previously presented near-threshold cue, the authors found an increase in prestimulus alpha performance on perceived versus unperceived trials. Linkenkaer-Hansen et al. (2004) reported that increased prestimulus power over parietal regions and intermediate power over sensory cortices were associated with an increased likelihood to detect near threshold somatosensory stimuli. Explanations for the complex and apparently conflicting data are explained away in the literature with differences in the task [memory process more or less involved, cf. discussion in Hanslmayr et al. (2007)] or with biophysical conditions [mixing of different processes at the electrodes due to volume conduction, cf. discussion in van Dijk et al. (2008)].

Because of this unclear data situation, there are now increasing voices suggesting a rethinking of long held and entrenched assumptions about the nature of the (possible) relationship between brain oscillations and cognition. For example, in a recent special issue devoted to clarifying the long-standing debate about whether and how brain oscillations are involved in cognitive processing, about half of the studies (eleven of 23) confirmed a link between brain oscillations and cognitive processing, while the other half did not. The editors of the special issue felt compelled to encourage the pursuit of new perspectives and the avoidance of simplifications in hypothesis generation (see Keitel et al., 2022). While latter referred specifically to the relationship between brain oscillations and cognition (e.g., influenced by entrainment), similar considerations apply equally to the modulation of prestimulus α-power: following a literature review, Iemi et al. (2017) point to several null-results, particularly for studies involving discrimination tasks, compared to studies focusing on detection alone. Moreover, based on their own experiments and inspired by signal detection theory (SDT), the authors suggest that reduced α-power before the stimulus reflects a liberal detection criterion rather than an improvement in sensitivity that would, eventually, lead to better accuracy in discrimination tasks *per se* (for a recent review, see also Samaha et al., 2020). This is an attractive idea to narrow the existing explanatory gap, but nonetheless open questions remain. For example, both reviews (Iemi et al., 2017; Samaha et al., 2020) omit studies examining prestimulus modulation of α-power in the context of TOJs from their considerations. However, we believe that the inclusion of studies examining TOJs is important because determining the temporal order of stimulus pairs, particularly around the individual temporal discrimination threshold, is also a discrimination task. Or put another way, an ambitious theory that seeks to explain the modulatory dynamics of prestimulus performance between detection and discrimination tasks would definitely benefit from including studies that examine prestimulus modulation in the context of TOJ tasks. Be the findings, on the face of it, as puzzling as they may be.

This imbroglio of results prompts questions. What emerges from the literature to this point is that prestimulus α-power and its (direction of) modulation appear to be both highly individually variable and context dependent. For example, the α-gating effect is generally present only in participants with high α-output values (e.g., Rihs et al., 2009). Also, experimentally induced modulation may vary greatly across participants in terms of localization or may even be absent in some participants (especially in auditory cortex; cf. Bastiaansen and Brunia, 2001). Not all individuals may have a pronounced α-peak (Grabot et al., 2017) and the frequency of individual α-peaks may vary (Haegens et al., 2014). Hanslmayr et al. (2007) discusses a dependence on the cuing condition and influences of associated memory processes (see also Klimesch et al., 2006). Slagter et al. (2016) reported that ipsilateral inhibition of irrelevant neural networks did not occur when participants were asked to keep their attention consistently on one side. The authors view this as evidence that modulation of α-power as an inhibitory top-down control process is only necessary when irrelevant networks actively compete with relevant networks for limited attentional resources.

Other (unknown) influences contributing to the inconsistencies observed in the literature could be caused by different exclusion decisions in the selection of data considered for the final analysis. For example, individuals with “exceptionally high alpha power-values (>3 SDs from mean)” were excluded (van Diepen et al., 2015). Excluding individuals without a pronounced α-peak from the analyses (e.g., Babiloni et al., 2006) could bias the results [see similar discussion in Love et al. (2013) in the context of audiovisual timing studies].

We find it remarkable that often no particular information about individual modulation direction is given, let alone discussed. The focus is mostly on group-effects. This is understandable in that group-effects allow generalization to the population as a whole (i.e., at best, to all humans). However, efforts to understand individual modulation direction are of particular importance when interindividual variability results in some individuals even act in the opposite direction of the expected effect (cf. Cohen, 2017). In such a case, relying on a “significant” group-effect is not sufficient for generalizability and understanding of a phenomenon. Individuals that run counter to a group-effect may contribute to null effects based only on the prevailing sample.

The importance of considering individual-level outcomes is exemplified by the study of London et al. (2022), who commendably show individual-level data (which is still an exception and not the rule). London et al. (2022) report that higher prestimulus α-power is associated with poorer (temporal) discrimination performance for audiovisual cues. Now, it is interesting to note that this is not true for all cases, but “only” for 85% of cases (if at all). In other words, some individuals show no effect or even an opposite effect [figure 3C shows that the latter is true in six out of 40 cases, and at least an equal number of other cases suggest a null effect (London et al., 2022)]. It would be bold to condemn (>) 15% of the cases as outliers or noise. The question, then, is what these individuals opposing the group-effect mean for generalizability and hypothesis generation. Especially when one considers that individuals *without* a (pronounced) α-peak or with increased α-power before the stimulus are also capable of (temporal) discrimination. We believe that it is a vain endeavor to establish a generalized theory without accounting for this variability at the individual level (see also Wriessnegger et al., 2020 for similar arguments). A better understanding of prestimulus dynamics in TOJs seems particularly informative to us, given that the data examining prestimulus modulation of α-power and its relationship to veridical TOJs from the three studies we are aware of are, on the whole, particularly puzzling: first, we have the Grabot et al. (2017) study mentioned above, which observed an association of veridical audiovisual stimulus pairs with attenuation of prestimulus α-power in auditory cortex and nowhere else (as also noted, such a pattern is rarely observed in the context of modulations of prestimulus power). Second, in a recent study (from the special issue mentioned above), London et al. (2022) reported that lower prestimulus-α activity in a posterior cluster was associated with veridical audiovisual TOJs. However, contrary to Grabot et al. (2017), they found no systematic association with central electrodes (i.e., over auditory brain regions), which would have been expected in the case of a pronounced involvement of auditory networks. Third and finally, Bernasconi et al. (2011) examined unimodal spatial TOJs with auditory stimuli (i.e., in each trial, two temporally offset auditory signals were presented on different sides, and participants were asked to judge on which side they perceived the first signal) and reported that veridical trials were associated neither with a downregulation of α-power before the stimulus in auditory cortex nor with an upregulation in any of the other but irrelevant sensory modalities (as would have been predicted by the active gating hypothesis). Rather, upregulation was observed only in the left posterior sylvian regions. However, this was not true for α- but for β-power. This finding contributes to the variability and puzzling nature of the findings in several ways: first, it contradicts what AIH would expect in terms of modulation direction; second, with the effect in the β-band, the modulated frequency in the prestimulus period is higher than is usually observed in detection or discrimination tasks with an α-band modulation. We are not aware of any studies investigating visual TOJs.

In summary, this mosaic of findings on the modulation of prestimulus power remains puzzling, and no overarching explanation has yet been provided. These seemingly contradictory data and the focus on group-effects, as briefly reviewed above, remind us of the long-standing debate in the literature as to whether individuals generally perceive the auditory or the visual stimulus as relatively faster. This literature includes studies claiming that one signal is perceived faster and studies finding the opposite. Different researchers have proposed different explanations, and in a similar way to the literature on the modulation of prestimulus power, the different results have been explained, for example, by differences in task design. However, we have shown (Boenke et al., 2009), on the one hand, that there is no “typical” pattern (at least not in the way it has been discussed), but that individual differences and the magnitude of the effect depend on complexity (i.e., uncertainty). Importantly, this influence of complexity is not apparent in the group-effect (average). On the other hand, we raised the question of how meaningful a question about the (relative) speed of perception of a signal with the tasks used is in the first place. It is by no means guaranteed that the task measures what the experimenter believes it measures. We believe that the unexplained variability between studies (seemingly contradictory results, but also null effects) and between individuals [as we exemplified with the London et al. (2022) data, but also to a lesser extent occurring in e.g., Foxe et al. (1998) and Fu et al. (2001)] requires further clarification. The latter is especially true in light of the ongoing replication debate in neuroscience, which calls for scrutinizing previously implicit assumptions and more frequent retesting (see Cohen, 2017; Keitel et al., 2022).

To this end, we performed a spatial TOJ task with randomly interleaved auditory and visual stimuli while recording participants’ scalp EEG. The design we used offers several advantages and new avenues of insight. Spatial TOJs allow us to present the auditory and visual stimuli within a session without having to rely on cues or switch between tasks. As suggested by Slagter et al. (2016), competition for limited attentional resources between relevant and irrelevant networks is required to trigger modulation of prestimulus α-power. However, cues resolve this competition, which may explain why no interaction between modalities in the direction of prestimulus modulation predicted by AIH has yet been demonstrated. Thus, cue omission may be the critical factor in demonstrating this interaction. In addition to testing the interaction between auditory and visual modalities predicted by the AIH, we can use auditory TOJs to test how robust the results of Bernasconi et al. (2011) are with respect to modulation direction (upward or downward) and frequency (α or β). Visual TOJs have not been tested before, this extension allows us to determine whether the TOJ itself has different modulation dynamics than less complex discrimination tasks. Involving the auditory and visual modality with an identical task (TOJ) within the same session also allows us to characterize the relative dynamics of prestimulus activity in each modality and as a function of task in a direct comparison. It will be interesting to see whether visual TOJs, if the results of Bernasconi et al. (2011) hold, also show an opposite result to the prevailing literature, or whether prestimulus dynamics in (spatial) auditory TOJs turn out to be a “special case” (in the sense that the level of complexity is even higher than for simple discrimination, let alone detection tasks). Finally, inspired by our previous work on audiovisual timing mentioned above, we have a special focus on individual variability to determine whether an emerging group-effect is homogeneously carried by the sample or whether there are individual-level manifestations of the effect that counteract the group-effect. Addressing these questions may contribute to reconsidering long-held assumptions about the context in which variability is noise or signal, and the extent to which generalizability of (dichotomously formulated) research questions is meaningful. They may also provide clues to a better understanding of variability across studies, which is important beyond the study topic itself.

## 2. Materials and methods

### 2.1. Participants

Our goals were to test the robustness of Bernasconi et al.’s (2011) results, to extend the tests to the previously unstudied visual TOJ, and to determine whether and to what extent a cue-free study design would be able to elicit an interaction between activity in the relevant modalities when employing auditory and visual stimuli (cf. Foxe et al., 1998; Fu et al., 2001). In the studies by Bernasconi et al. (2011) and Foxe et al. (1998) we found relevant information that we could use to estimate the required sample size. With a sample of *N* = 11, Bernasconi et al. (2011) report a high t-value for their effect (difference in global power spectra at 20 Hz): “[t(10) = 8.663; *p* < 0.02; ηp2 = 0.464],” indicating a very strong effect [similar to the study by Foxe et al. (1998) with a sample of *N* = 12 and a reported t = 3.423 for the increased α-power at parieto-occipital sites when attention was directed to the auditory modality]. Based on this presumed very strong effect in the studies relevant to us, we conservatively assumed an effect size of Cohen’s d = 1 as a first approximation. From this starting point (d = 1, *N* = 11), we calculated a critical t-value (t = 2.23) and sufficient power to detect an effect (85%) using G*power (Faul et al., 2009). We chose to use a larger sample of *N* = 16 from which we can expect an increase in power to >95%.

In the end, we recruited a total of twenty paid volunteers, as we had to exclude data from four participants before further analysis due to either poor channels in the region of interest (two), lack of triggers (one), or hardware communication problems (one). The targeted remaining 16 participants (age range 20–28 years; mean age 24.1 ± 0.55 s.e.m.; eight women, 15 right-handed, one two-handed based on self-report) had normal or corrected-to-normal visual acuity and normal hearing abilities. Participants gave signed informed consent. All procedures conformed to the Declaration of Helsinki.

### 2.2. Apparatus

All experiments were performed in the same, sound-attenuated, electrical shielded chamber and experimental setup as described in earlier work (Boenke et al., 2009). The chamber had an ambient sound level of ∼28 dB(A) SPL and the setup was covered with black velvet cloth to avoid light reflections. A green light-emitting diode (LED) at eye level ∼165 cm from participants served as fixation. Two boxes were placed symmetrically ±38 cm left and right of the LED (∼ ± 13.0° visual angle), each containing a speaker with a white cover and a white LED light source mounted above the speaker inside the box. A 4 cm aperture in the front of the box revealed the speaker cone, and the white cover in front of it would appear as a white circle whenever the overhead LED was illuminated and was otherwise invisible. This set-up allows presentation of collocated light and sound sources with independent timing. Stimulus presentation and recording was controlled by a Matlab (R14) program running on an IBM 486-compatible microcomputer and participants responded on a custom-made hand-held response box. Stimulus timing was controlled by a National Instruments card (PCI-6071E, Austin, TX, USA) and was verified to be accurate to <1 ms.

### 2.3. Stimuli and experimental design

Stimuli and design were followed our earlier psychophysical work (Boenke et al., 2009) with the difference that we here focused on unimodal conditions and employed only one stimulus duration (9 ms) and fewer stimulus onset asynchronies (SOAs) to allow a higher number of repetitions per observation for the electrophysiological analysis. We used a spatial TOJ task using the method of constant stimuli and randomization without replacement. By a spatial TOJ task we mean that the stimuli appeared either side of fixation with asking participants to judge on which side they have perceived a signal onset first. A spatialized task has several advantages: By asking participants to report which side they first perceived a stimulus onset, the task is orthogonal to stimulus modality. There is evidence this design reduces bias to a specific modality (Shore et al., 2001; Spence et al., 2001) and, importantly, it allows us to mix auditory, visual and audiovisual trials within one session without changing task instructions or cues. It also prevents potential imbalances between modalities due to the choice of cue-type and modality [cf. Foxe et al., 1998; Fu et al., 2001; and as discussed in Foxe and Snyder (2011)].

Stimuli for the auditory TOJ (AA) were white noise bursts with 2 ms onset and offset ramps and an intensity of 50 dB(A) SPL. The LEDs delivering the stimuli for the visual TOJ (VV) had a rectangular temporal profile and an intensity of 0.64 cd/m^2^. For technical reasons, luminance and sound pressure level (measured at participant’s head position) were obtained for stimuli with duration of 5 s. All stimuli were for all participants clearly above threshold. There was also an audiovisual (AV) TOJ condition which involved one auditory and one visual stimulus within a trial as described above. Besides that the inclusion of AV trials was intended to further motivate participants to spread their attention across both modalities it also addresses a different experimental question not treated in this report and consequently will not be considered further here. Our design involves a slight deviation from that employed by Bernasconi et al. (2011): In their report the EEG study was preceded by a psychophysical test to measure auditory TOJ thresholds for each individual which allowed them to present single individually adjusted SOAs. Since we presented stimuli in three 3 contexts TOJ conditions (auditory, visual, bimodal) we expected also, as previous research showed [e.g., Zampini et al., 2003; reviewed in Keetels and Vroomen (2012)], potentially three different temporal order thresholds (TOT). Although it is in principle possible to pretest the threshold for each condition and then present only the SOAs at specific thresholds [as in Bernasconi et al. (2011) for their auditory-only condition], we believe that presenting the same SOAs for each condition and identifying the SOAs near the threshold post-hoc is the better strategy for our purposes: first, presenting three different SOAs (between conditions) but always the same SOAs (within conditions) could lead to temporal inconsistencies between modalities (Van der Burg et al., 2013). Second, the under complexity of repeated presentation of the same stimuli could cause learning effects to occur more quickly (making the task simpler), and, further complicating matters, possibly at different rates for each condition. Apart from this, it also allowed us to analyze the TOTs with a more complete representation of a psychometric function, facilitating comparison with other (behavioral) TOJ studies.

In total the experiment involved 1,440 trials [3 SOAs (90 ms, 55 ms, 20 ms) × 2 stimulus first (A/V) × 2 side (left/right) × 2 modality (bimodal, unimodal) × 60 repetitions] organized into six experimental blocks of 240 trials (yielding five repetitions for each observation per block). Unimodal includes AA and VV conditions and bimodal includes AV (A first) and VA (V first) conditions. Each condition consists of 360 trials. Thus, unimodal and bimodal trials are not only perfectly balanced numerically, but this is also true for the number of trials in which a particular modality was presented first (in terms of number, the following applies: AA = AV, VV = VA, AA + VV = AV + VA).

### 2.4. Procedure

Participants’ task was to give an unspeeded report on which side they perceived the first stimulus onset using the corresponding left or right button on custom made response box emphasizing accuracy. Participants were asked to give their best guess when uncertain about the perceived order. Trials were self-paced. To minimize the development of a temporal expectation of the next trial onset, each response was followed by an intertrial interval of 1.5 s – 2 s duration randomly selected from a rectangular distribution. The green fixation LED was illuminated throughout the experiment and participants were instructed to (1) maintain their gaze on it and (2) align their face toward it while avoiding horizontal head movements during trials (initiated after a report by button press). Each experiment was preceded by a training block corresponding to one of the six aforementioned blocks of the actual experiment. Apart from checking and ensuring that veridicality was present in more than 60% of trials (across all SOAs), which was the minimum requirement for participation in the main experiment, the training blocks were not analyzed further. All remaining 16 participants met this minimum requirement. Depending on the block and participant, each block lasted between 11 and 15 min.

### 2.5. Data acquisition and preprocessing

#### 2.5.1. EEG data

Scalp EEG was recorded from 62 Ag^+^/AgCl^–^ electrodes, placed according to the international 10/10 system, mounted in an electrode cap (M 11, FMS, Herrsching, Munich, Germany), and using a high input impedance amplifier (10 MΩ, BrainAmp, Brain Products GmbH, Gilching, Munich, Germany). Eye-blinks and -movements were monitored using two additional electrodes placed below and next to the right eye. Electrodes AFz and FCz were used as ground and physical reference, respectively. Before data acquisition electrode impedances were reduced below 5 kΩ. Data were digitized at 1,000 Hz and recorded with 0.1 Hz high-pass and 100 Hz low-pass filter. Data were subsequently re-referenced offline to average reference. To ensure a direct comparison with the work of Bernasconi et al. (2011), we only consider epochs of EEG signals 200 ms before stimulus onset in our analyses. The DC components were removed and further artifacts rejected if they exceeded the pre-set threshold of ±100 μV which also served, together with visual inspection, as eye-blink removal. Across all participants, artifact rejection and visual inspection resulted in an average trial reduction of (10.72 ± 2.27)% (mean ± s.e.m.) in AA and (10.05 ± 2.01)% in VV. Trial reduction was evenly distributed across modality and veridical/non-veridical conditions.

By testing auditory and visual TOJs using identical tasks within the same session, we can additionally characterize the relative dynamics of prestimulus activity in each modality and as a function of task (cf. section “1 Introduction”). To do so, we needed to compare veridical against non-veridical trials around the TOT for each condition (AA and VV) and then examine the correlation with prestimulus dynamics preceding each event. For the analysis comparing brain states between modalities, trials with events presented “left-first” were collapsed with events “right-first” (there was no difference in support of a two-tailed paired *t*-test: TOJ AA *p* = 0.6 and TOJ VV = 0.3; see section “3 Results and discussion” below). This also increased number of trials for each condition for an SOA and modality per participant from 60 to 120. Due to our strategy to interleave and employ a common set of SOAs for all conditions not all SOAs were singly around the TOT as in the main experiment in Bernasconi et al. (2011). Instead, we chose corresponding SOAs post-hoc. This was achieved by selecting for each participant and each condition separately the SOA being closest to the pre-defined level of veridicality of 75% which is commonly used in the literature (cf. Keetels and Vroomen, 2012). Selecting a pre-defined TOT closest to the level of 75% naturally introduced different numbers of veridical and non-veridical trials (theoretically 90 veridical and 30 non-veridical with in total 120 trials). To balance this effect and because of the overall good performance in the VV condition the SOA for two participants was chosen to be at least >60%. Next, to control for signal-to-noise ratio, the number of veridical and non-veridical trials were matched by random pick operation from the condition with more trials (cf. Bernasconi et al., 2011). Importantly, since we were interested in the prestimulus dynamics this strategy using different SOAs did not introduce any imbalance in the signal of interest (namely prestimulus epoch).

Based on this preprocessing, we obtained for the modality comparison on average 32.50 ± 1.86 (mean ± s.e.m., range 22–49) trials for AA, and 32.69 ± 3.09 (range 12–49) trials for VV (no statistical difference).

#### 2.5.2. Short-time fourier transformation (STFT) and further EEG data analysis

For all conditions (AA-veridical, AA-non-veridical, VV-veridical, VV-non-veridical) and all participants separately, we calculated three spectral components from 10 Hz to 20 Hz (5 Hz steps) using STFT with a time window beginning 200 ms prior to the onset of the first stimulus similar to Bernasconi et al. (2011). Given that functionally similar frequencies may not necessarily have stationary frequency values across experiments or individuals (e.g., for α, see Chiang et al., 2008), we did not average frequency components into traditional frequency bands or arbitrary bins but considered specific frequency values instead. This approach minimizes the likelihood of averaging across functionally different oscillators. Finally, we calculated Power Spectral Density (PSD) for all epochs and for each frequency component, participant, TOJ condition (AA, VV), and response (veridical vs. non-veridical) separately. Next, for comparison with the results in Bernasconi et al. (2011), we contrasted the global power spectra (GPS, i.e., the average value of the frequency power at each electrode) between veridical and non-veridical trials for the AA TOJ condition (and subsequently for VV TOJ).

#### 2.5.3. Standardization (studentization) and cross-modal prestimulus dynamics

Since in GPS local information is lost, it is not adequate for our second goal, i.e., studying the dynamics of the relative prestimulus brain states between auditory and visual modality when one of the modalities is considered relevant and the other irrelevant for upcoming stimulation (cf. Foxe et al., 1998; Fu et al., 2001). To retain local information, we studentized frequency values across the 62 sensors for each condition (AA, VV), frequency (10, 15, 20 Hz) and participant. This approach not only takes global information into account but also assigns each sensor with a specific standardized value expressing its activation level relative to the global activation. This procedure has several advantages. The normalization of each frequency allows direct comparison of different dynamics at each frequency, as the studentized values reflect the relative performance at a given sensor within a given condition, thus normalizing the known (supra) exponential power decline with increasing frequencies (e.g., Cohen, 2017). Moreover, this approach normalizes the difference in absolute α-power between the two modalities: while the globally highest measurable α-power is measured over parieto-occipital areas, it is about one magnitude weaker over central ones. Such a difference may mask the relative shift in power between modalities and which may have contributed to the emergence of a general debate about the existence of auditory α-power (cf. Weisz et al., 2011) and made it difficult to be detected in earlier studies (Foxe et al., 1998; Fu et al., 2001). Last but not least it normalizes individual differences of α-power baseline levels across participants which are known to be large and can have an impact on the results of studies of this type reported here (e.g., Chiang et al., 2008; Rihs et al., 2009).

### 2.6. Statistical analysis

**FIGURE 1:**
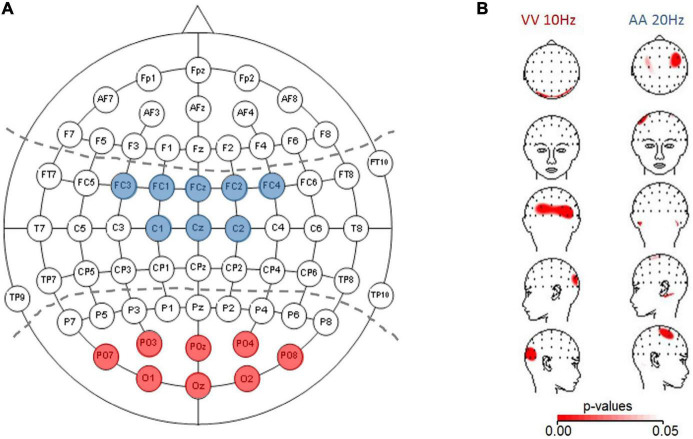
**(A)** ROIs depicted for analyzing auditory activation (central electrodes, blue) and visual activation (parieto-occipital electrodes, red). **(B)** Topographic plots (using spherical splines of *p*-values < 0.05 in red) for those conditions which are within the a/b-band and fulfilled the a *priori* criterion that at least three neighboring electrodes must be significant. Left panel: visual (VV) condition (10 Hz) with higher activation (based on power spectral density) over parieto-occipital electrodes for veridical response trials compared to non-veridical response trials. Right panel: auditory (AA) condition (20 Hz) with higher activation over auditory electrodes in veridical compared to non-veridical trials.

For statistical evaluation of frequency-specific activation dynamics in auditory and visual modalities, we defined ROIs following the electrodes typically chosen in auditory or visual tasks in the literature (partially adapted to our different cap layout; see Figure 1A for the predefined electrodes for each modality (e.g., Fu et al., 2001; Sauseng et al., 2005; Hanslmayr et al., 2007). The average across the electrodes within a specific ROI was fed into a three-factor ANOVA: Frequency (10, 15, and 20 Hz) × TOJ condition (AA and VV) × ROI (A-pool and V-pool).

#### 2.6.1. Correlation analysis

By using a spatial TOJ task we were able to test participants in auditory and visual conditions (AA, VV) without any task switch enabling us to study, first, if α-band modulation is consistent across participants over both conditions (sign of relative activation in auditory and visual modality in AA is correspondingly reversed in VV) and second if the magnitude of the effect is consistent (e.g., large effects in AA are also large in VV etc.). To test the first point, we plotted difference standardized values for each participant in the V-pool over those obtained in the A-pool, for each condition separately. Since standardized values reflect the local activation relative to global activation, we expect by definition that high values at some electrodes must be accompanied by low values at others (as globally there must be a zero sum). If there was a dynamic shift between modalities as predicted by the AIH, we would expect in the AA TOJ condition that standardized values in the A-pool should be relatively low and in the V-pool relatively high (A-pool < V-pool), and *vice versa* for the VV TOJ condition (A-pool > V-pool). To test the second point, that is if α-band modulation is consistent across participants, we again plotted the V-pool over the A-pool but took for each pool the ‘incongruent minus congruent’ difference TOJ. That is, for the V-pool we calculated the standardized values obtained in AA TOJ minus the standardized values obtained in VV TOJ, and for the A-pool the standardized values obtained in VV TOJ minus the standardized values obtained in AA TOJ. If the effect is similar in magnitude across conditions, we expect those participants with a large effect (in the sense of the AIH) to get relatively more positive values, those with a small or null effect to be around zero and, finally those with opposite effect (of AIH) relatively more negative values, producing an overall positive correlation. The more extreme a value is the larger is the (consistent) effect and magnitude of relative shift across modalities.

#### 2.6.2. Behavioral data

Behavioral data were analyzed in two ways. First, we averaged for each TOJ condition (AA, VV) across the SOAs depicted for each participant (see section“2.5 Data acquisition and preprocessing”). Second, the three different SOA values (20, 55, 90 ms) tested for each participant enabled us to estimate both the point of subjective simultaneity (PSS) and the TOT derived from more completely represented psychometric functions facilitating comparison of our approach (based on selecting the SOA from the three values that were associated with the veridicality score closest to 75%) with other TOJ studies. Therefore, we calculated the proportion of “right-first” responses for each SOA and performed a probit analysis after Finney (cf. Finney, 1964). In case of saturated response levels (e.g., proportion “right-first” responses of 0.0 or 1.0) values were substituted by 0.001 or 0.999, respectively. The PSS was defined as the abscissa-value where “right-first” and “left-first” responses were equal (y = 0.5, 50% of veridical responses) and the TOT was derived by taking the mean of the estimated absolute abscissa-values at 25% and 75% veridical responses (cf. Keetels and Vroomen, 2012).

## 3. Results and discussion

### 3.1. Behavioral results

As outlined in “2 Materials and methods” we chose for each participant and condition those SOAs closest to 75% veridical responses (mean percentage of the chosen SOAs (mean ± s.e.m.): AA = (72.72 ± 0.02)%, range: (60.83–87.50)%; VV = (71.17 ± 0.02)%, range: (58.33–89.17)%) assuming them to reflect the temporal threshold. From these performance values we obtained mean SOA values of 59.4 (±6.29, range 20–90) ms for the TOJ AA and 39.7 (±5.51, 20–90) ms for the TOJ VV condition. There was a difference between both conditions with a lower TOT for VV compared to AA condition [unimodal paired *t*-test: [t(15) = ±3.05, *p* = 0.024]. Using the traditional way of estimating PSS and TOT across all SOAs using probit analysis (Finney, 1964; see also Zampini et al., 2003) we found TOT to be 68.0 (±12.0, range: 25.3–195.9) ms for AA and 42.4 (± 5.1, range: 22.3–105.5) ms for VV, confirming the result obtained by picking SOAs around a pre-defined threshold that AA TOT was higher than VV TOT [t(14) = ±2.21, *p* = 0.045]. One participant could not be fitted due to low performance and were not considered in this more traditional behavioral analysis (and comparison between both conditions, see below). However, this had no impact on our SOA picking strategy for our electrophysiological results, since in this approach no fitting is necessary.

Slightly lower TOT values using the probit analysis are expected because the ordinate-values selected in our method were on average slightly below 75%. Correlation of TOT values based on our method with those obtained by probit analysis further justified our strategy [spearman rank test: r = 0.89, *p* = 0.00001 for AA (*N* = 15), r = 0.74, *p* = 0.001 for VV (*N* = 16)]. Moreover, TOT values in this study are very similar to those reported in other studies using also free field spatialized TOJ tasks and a similar method for estimating psychometric functions [e.g., 40.6 ms in Experiment 2 of the study of Zampini et al. (2003) for VV and 59.3 ms for AA]. However, auditory and visual TOTs were not significantly correlated suggesting at least partly independent factors in performing those tasks.

Probit analysis also allowed us to estimate the point of subjective simultaneity (PSS) for right- and left-first responses in each condition. We found the PSS to be 3.1 (±6.6, range: -37.0–52.2) ms for AA and -5.1 (± 4.6, range: -35.2 to 40.7) ms for VV (with negative values meaning left stimulus needed to be presented first for reported simultaneity). Between modalities, group mean PSS were not statistically different from the expected value of 0 ms [AA: t(15) = ±1.31, *p* = 0.45, VV: t(15) = ±0.78, *p* = 0.21].

**FIGURE 2:**
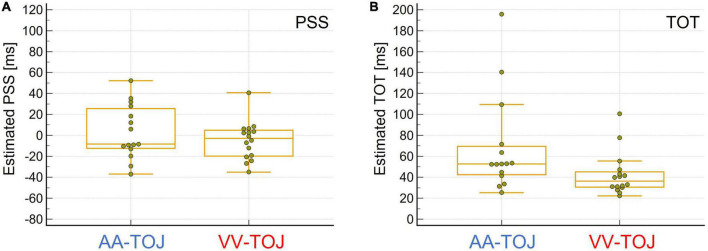
**(A)** Boxplot for estimated point of subjective simultaneity (PSS) values, i.e., when stimuli presented in pairs (one from the left, one from the right or vice versa) were judged to be simultaneous (estimated highest uncertainty about order). Auditory (AA)-temporal-order judgment (TOJ) (*N* = 15, left, blue) and visual (VV)-TOJ (*N* = 16, right, red). Negative PSS values indicate that the physical onset of the left stimulus must precede the physical onset of the right stimulus to be judged as simultaneous, whereas positive PSS values indicate that the physical onset of the right stimulus must precede the onset of the left stimulus. The group means in both conditions were not different from 0 ms. However, PSS values are more scattered in the AA-TOJ condition than in the VV-TOJ condition. See text for further explanation. **(B)** Box plot of the estimated sensitivity of how well an individual was able to temporally discriminate the paired stimuli presented from left and right (temporal order threshold, TOT). AA-TOJ (*N* = 15, left, blue) and VV-TOJ (*N* = 16, right, red). Individuals tended to be less sensitive in the AA-TOJ condition than in the VV-TOJ condition. Note the greater scatter in the AA-TOJ condition compared to the VV-TOJ condition. See text for further explanation.

In summary, different thresholds in different modalities and the fact that performance in one modality was not a predictor of performance in the other modality (as indexed by rank-correlation of participants’ performance using SOAs/TOTs between modalities) challenge the notion that TOJs can be exhaustively explained by a single central comparator in a simple way (e.g., Sternberg and Knoll, 1973). The lower sensitivity of AA compared to VV seems unusual at first sight and contradicts the general finding that auditory modality usually has advantages over visual in the temporal dimension. However, since the task posed has a spatial component (and the visual modality has clear advantages over the auditory modality here), this result can be well-explained. Interestingly, the lower sensitivity of AA (indicated by higher TOT) compared to VV is accompanied by a higher variability of PSS scores and TOT (Levene’s test suggests that the assumption of equal variance for the TOT between the AA and VV conditions could not be supported: AA > VV: F = 4.5, *p* = 0.04; the differences in the PSS scores showed a trend in the same direction: AA > VV: F = 3.2, *p* = 0.08; see Figure 2) between participants in AA compared to VV which is also consistent with the study by Zampini et al. (2003), but was neither tested nor discussed further there.

This difference in variability between conditions is not trivial, as everything except the different stimulus dimension was identical between the two conditions (experimental set-up, participants). Lower sensitivity is equivalent to higher (sensory) uncertainty. That higher uncertainty leads to higher variability we have already discussed in Boenke et al. (2009) in the context of audiovisual TOJ, where also higher uncertainty (in terms of sensory noise at shorter stimulus duration) led to higher variability between participants (Boenke et al., 2009, 2014) and what we attributed to the relative occurrence of intrinsic strategies (more generally: states). In other words, under (sensory) uncertainty, one of the possible states is realized randomly, whereas under lower (sensory) uncertainty, realization occurs according (or is constraint) to the physical situation.

The difference in TOT shows two “outliers” for each of the modalities (Figure 1B). These outliers can be attributed to the individual relatively poor fits in the probit analysis, and it might therefore be questioned to what extent this pattern of differential variability in AA and VV TOJ is reliable. Importantly, however, the poor fits are always associated with correspondingly poor discrimination performance. The worse the performance, the worse the fits (recall that one data set for AA TOJ could not be fitted at all due to very poor discrimination performance and thus does not even contribute to the distribution shown in the figure), which also means that the differences between modalities can be considered “real” for these outliers and thus for the entire distribution of individual data.

Another interesting aspect is the scatter of PSS values (Figure 1A). A few individuals deviated relatively far (>20 ms, i.e., the smallest measured SOA) from the expected group-effect of 0 ms in one direction or the other (some individuals inclined to judge the left stimulus as first, others inclined to judge the right as stimulus first). However, we did not find this pattern of behavioral data to be clearly and systematically reflected in the EEG data (prestimulus power, activation side) reported and analyzed here. Nevertheless, as mentioned in the Introduction, this pattern of results raises the general and important question of the extent to which a group effect can and should be generalized to the individual.

### 3.2. Electrophysiological results

#### 3.2.1. Global power spectra (GPS) and *t*-tests for veridical versus non-veridical trials

Because a major concern of our study was to see how robust the results of Bernasconi et al. (2011) were, we followed their analysis strategy and compared the GPS of veridical with non-veridical trials (first in AA, then for VV). We found that the numerical GPS values for most conditions and frequencies were larger on average for veridical trials than for non-veridical trials. However, this difference was in neither condition supported by a test (paired bidirectional *t*-tests, Bonferroni-corrected). Examining participant-level GPS scores for the auditory (AA) 20 Hz frequency [reported as significantly different in Bernasconi et al. (2011)], 13 participants showed veridical >non-veridical GPS. That is, considering the GPS as an index of higher global activity, only 3/16 participants agreed with the AIH [or 13/16 with the mean reported in Bernasconi et al. (2011)]. If we use the threshold for outliers commonly used in the literature (2 SD deviation from the mean), a participant could be classified as an outlier who is more than 2 SD smaller than the mean of all participants (deviation in the direction predicted by the AIH). Exclusion of this participant had a clear impact on the test result and replicated the report of Bernasconi et al. (2011) that the AA 20 Hz frequency band had a higher GPS for veridicality (*p* = 0.0046). Be that as it may, we are critical of the exclusion of participants with such a “blind” (i.e., ritualized and not motivated by e.g., theoretical considerations) criterion as 2 SD for several reasons. Moreover, we do not find it so remarkable that by excluding a so-called outlier we were able to push our *p*-value below the “magic” threshold of 0.05, which at first glance suggests that only by doing so we are in agreement with Bernasconi et al. (2011), because even this does not change the, from our point of view, more remarkable fact that several individual data did not agree with the sign of the mean and thus contradict the group effect (raising the question to what extent the question of a specific modulation direction is meaningful). We view the exclusion of an outlier that has such a dramatic effect on the “significance” of the result rather critically and as a warning of how easy it can be to push a *p*-value below a certain threshold (or, conversely, to miss a threshold by adding a data point; cf. p-hacking: Simmons et al., 2011). However, exclusion comes at a cost: variability is reduced, making the remaining sample more homogeneous. Ultimately, one inadvertently obstructs the path to interpreting variability as a potential signal, rather than dismissing it as noise, as is usually the case (although this is slowly but surely changing, cf. recent review articles: e.g., Seghier and Price, 2018; Waschke et al., 2021). Increasing homogeneity of the sample may favor group-effects but reduces power for correlation analysis (cf. Hedge et al., 2018). Thus, the exclusion of outliers can lead to a vicious circle that ends up reinforcing implicit assumptions (e.g., the previously assumed null hypothesis). In principle, with this result we already have a situation as described in the introduction: different results can be a mere result of the sample and individual data must be better understood. We will come back to this.

Next, we performed a paired *t*-test for veridical and non-veridical trials across all electrodes for the AA and VV conditions separately. The advantage over the GPS analysis is that we have access to the local information of the prestimulus power modulation between veridical and non-veridical judgments. It serves as a summary of all effects. It also allows us to identify between-electrode effects without the potential selection bias introduced - by observer dependent selection of ROIs. On the other hand, these advantages come at the price of increasing the likelihood of false positives. To balance this, we set a spatial constraint to reduce the likelihood of a false positives by considering as “real” only those effects where at least three neighboring electrodes are below a *p*-value < 0.05. Although there is currently no objective criterion for how many neighboring electrodes must be below the specified threshold, there are examples in the literature of using a similar strategy (e.g., Murray et al., 2004; Barcellona-Lehmann et al., 2010). Most importantly, these tests were not conducted as a hunt for “statistical significance”. Rather, they served as a comprehensive overview of our data. This did not take place in a vacuum and was guided by a prior and clear expectation/hypothesis that we established on the prior work of other authors.

The results showed that in the AA condition, only 20 Hz showed a difference in three right neighboring central electrodes, with veridical trials showing higher prestimulus baseline power compared to non-veridical trials. It should be noted that on the left side the criterion was very narrowly missed due to one electrode (out of three) with *p* = 0.05. For the VV condition our analysis indicated effects in eight neighboring parieto-occipital electrodes for 10 Hz (across hemispheres), and in four electrodes over the right hemisphere in VV 15 Hz. All results reported above which fulfill our pre-set criterion designed to minimize spurious results were in line with Bernasconi et al.’s (2011) study showing opposite activation patterns than predicted by the AIH (veridical > non-veridical) and a modulation of prestimulus power in the β-band. However, as with the GPS not all individuals were in accordance with the group-effect. The prestimulus power modulation in the VV condition, besides that the modulation we found is in opposite direction, are evident at electrodes which are consistent with previous studies investigating prestimulus α-power modulation with detection or discrimination of visual stimuli (e.g., Foxe et al., 1998; Fu et al., 2001; Hanslmayr et al., 2007).

Figure 1B depicts *p*-values projected on topographic maps using spherical splines for the auditory 20 Hz and visual 10 Hz effects, demonstrating the expected topographies for the two sensory modalities. Interestingly, for both conditions, when plotting the difference map between veridical-non-veridical absolute power-values (not shown) in the same way as for the *p*-values, the greatest difference appeared on the left hemisphere, whereas the *p*-value topographic map showed a trend of the global extreme (in terms of smallest *p*-values) over the right hemisphere. This could be traced back to higher variability over left hemisphere compared to right hemisphere electrodes across participants, causing the *t*-tests to not reach significance. One explanation could be the known difference between the left and right hemispheres: Compared to the right hemisphere, the left is more infolded (which may influence the signal picked up on the scalp), has more gray matter, higher conduction delay (more dispersion of the signal in time), and shows a lower coherence between recording sites in wide range of frequency bands [reviewed in Miller (1996)]. Probably reflecting overall a signature of a trend of (temporally) more complex processing in the left hemisphere (as e.g., language processing) and demonstrates once again the importance of considering (often neglected) variability in the interpretation of means in neuroscience, and the importance of hypothesis-driven interpretations as opposed to automated testing.

**FIGURE 3:**
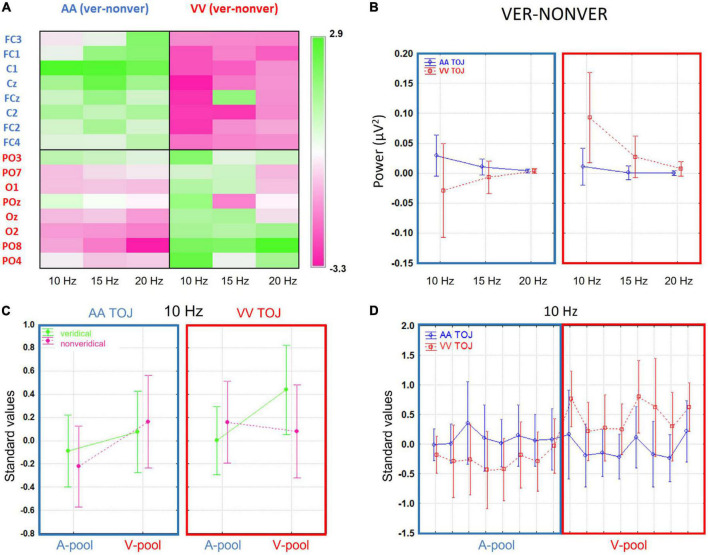
**(A)** The difference in absolute power-values of veridical trials minus non-veridical trials for both conditions [auditory (AA) temporal-order judgment (TOJ), blue lines; visual (VV) TOJ, red lines; error bars show 0.95 confidence intervals], all three investigated frequencies (10, 15, 20 Hz) and for the respective region of interest (ROIs) (A-pool, in blue; V-pool, in red). The expected (supra) exponential power decline with increasing frequency is striking. However, it is also particularly clear that the (absolute) difference between the conditions is especially pronounced at 10 Hz. Even more: there is an interaction between condition and veridicality. **(B)** Grand average channel-frequency plot of difference standardized values (veridical—non-veridical): Left panel for AA condition and right panel for VV condition. Green color denotes positive, that is higher activation for veridical than non-veridical trials, and pink colors negative, that is lower activation for veridical than in non-veridical trials, standardized values. There is a clear interaction between ROIs and condition is evident which is especially pronounced in the 10 Hz frequency. **(C)** Standardized values (10 Hz) broken down into veridical (green lines) and non-veridical trials (pink lines, mean ± 0.95 confidence intervals). Left panel indicates the means across all participants in the AA TOJ condition (blue box), right panel the VV TOJ condition (red box). A-pools are on the left and V-pools on the right within the boxes (reflecting the mean values across pools and 10 Hz in Figure 3A). In the V-pool all standardized values are positive irrespective of veridicality suggestive of an overall higher α-activity over this region compared to central regions. The differences between veridical and non-veridical judgments are for AA and for VV conditions especially pronounced over respective pools (AA, A-pool; VV, V-pool) and the average of prestimulus power was always higher than for non-veridical judgments. **(D)** Standardized values (10 Hz) in AA TOJ (blue line) and VV TOJ (red line; error bars depict 0.95 confidence interval) for all electrodes of our pre-selected ROI (A-pool, blue box; V-pool, red box) demonstrating a clear and consistent interaction.

Although activation levels at most electrodes tended to be larger on veridical trials compared to non-veridical trials, some local areas showed the reverse pattern. Moreover, even in areas where the group *t*-test difference was significant, there were single participants showing opposite effects (thus in line with the AIH hypotheses). We will discuss this in more detail in the section “4 General discussion”. Up to this point, our results resemble those of Bernasconi et al. (2011) with GPS levels and *t*-tests converging in a consistent way on an auditory effect over central electrodes at around 20 Hz. Moreover, we extended their findings to the visual modality and demonstrated a visual effect over parieto-occipital electrodes at 10 Hz (topographically widespread), overall implying a strong modality-specific influence of prestimulus power on TOJs veridicality. To better understand the differences between the AA and VV conditions in terms of frequencies suggested by the paired *t*-test summary, we plotted in Figure 3A the difference in absolute power-values of veridical trials minus non-veridical trials for both conditions (AA TOJ, blue lines; VV TOJ, red lines; error bars show 0.95 confidence intervals), all three investigated frequencies (10, 15, 20 Hz) and for the respective ROIs (A-pool, blue box; V-pool, red box). The expected (supra) exponential power decline with increasing frequency is striking. However, it is also particularly clear that the absolute difference between the conditions is especially pronounced at 10 Hz. Even more: There is an interaction between condition and veridicality. This we will further elaborate in the next section.

#### 3.2.2. Relative shifts of prestimulus activity between auditory and visual modalities

One of our goals was to study the relative shifts of prestimulus activity between auditory and visual modalities when comparing respective activations for veridical versus non-veridical trials in auditory and visual TOJs. The first step was to identify a coherent pattern across electrodes and frequencies (with around 10 Hz being typically thought to be relevant to the AIH). For this purpose, we plotted a sensor-frequency map for the depicted ROIs [cf. Figure 1A Blue labels denote the auditory ROI (A-pool) and red labels the visual ROI (V-pool)] as described in the method section for the standardized values of the mean difference values (veridical minus non-veridical) for the AA and VV TOJ conditions separately (Figure 3B). Upon inspection of the frequency map, several features were immediately apparent: for the central and parieto-occipital electrodes, the change in α-band power in the 10–20 Hz range varied in opposite ways for the AA and VV conditions and indicated a clear interaction between activity in the ROI and TOJ conditions in which the AA TOJ condition (left panel) has on average relatively higher local activation over central electrodes (A-pool) and relatively lower local activation over parieto-occipital electrodes (V-pool). The right panel of Figure 3B (VV condition) shows the opposite pattern, with higher standardized values over V-pool and lower standardized values over A-pool. Especially for 10 Hz, this dependence appeared to be consistent across nearly all individual electrodes of the ROI. This pattern suggests that the prestimulus modulation direction changes systematically according to task demands [or, to see it from a different perspective, that certain states (or strategies) are beneficial for better performance on a particular task]. That the prestimulus modulation is consistently pronounced, particularly in the α-band, is consistent with AIH. However, as already pointed out, we find for both the auditory and the visual TOJ on average across all participants a higher activation for veridical trials, which, related to the specific direction of the modulation, is apparently first of all in contrast to what the AIH predicts [but in line with the findings of Bernasconi et al. (2011)].

Visual evidence was verified by an interaction between the main factors TOJ condition × ROI [1, (15), F = 5.84, *p* = 0.03]. In Figure 3D we plotted the standardized values in AA TOJ (blue line) and VV TOJ (red line; error bars depict 0.95 confidence interval) for all electrodes of our pre-selected ROI (A-pool, blue box; V-pool, red box) demonstrating a clear interaction [15, (225), F = 2.2, *p* = 0.007].

Figure 3C plots group mean standardized values for veridical (green lines) and non-veridical trials (pink lines) for each condition (AA, blue box; VV, red box) and ROI (A-pool, V-pool). Figure 3C shows that the largest difference between A-pool and V-pool occurred on veridical VV trials, in which activation was increased over V-pool electrodes and decreased over A-pool electrodes. There was no such difference during non-veridical VV trials. This pattern was slightly different for AA trials. Here, there was no significant difference between A- and V-pool activation for veridical trials, instead, now non-veridical AA trials showed a higher activity over the V-pool compared to the A-pool. Overall, this pattern seems to suggest that visual veridical TOJs are associated with a relative shift of α-band power, whereas for auditory TOJs this relative shift is associated with non-veridical trials. However, comparing the relative shifts between veridical and non-veridical trials in the two conditions across the same pools suggests a common pattern across TOJ conditions: In AA and in VV conditions, the difference between veridical and non-veridical trials can be generalized as a relative increase of activation in the congruent pool and a relative decrease of activation in the incongruent pool of electrodes. This is opposite to the prediction of the AIH model and will be further evaluated below by examining the data on the participant level. Lastly, the local activation in each pool relative to the global activation shows positive standardized values in the V-pool (in each condition and performance level), indicative of an overlap with general higher α-power levels seen typically in parieto-occipital areas and independent of a specific task.

Interestingly, the pattern for all veridical trials (AA and VV) is very similar to that reported for “go trials” in a go/no-go paradigm by Foxe and colleagues (Foxe et al., 1998; Fu et al., 2001) in terms of an effect over parieto-occipital electrodes and no effect over central electrodes. Consequently, when considering only the veridical trials in our study (corresponding to successful go trials), all three studies report the same general pattern of results (apart from the direction of the modulation). Through our comparison of veridical and non-veridical trials, we also see that there is a power dynamic in the A-pool between the AA and VV conditions. This corresponds to the interaction that previous studies have missed to reveal, presumably due to the use of cues in their experimental design (see section “1 Introduction”).

#### 3.2.3. Participant-level analysis: correlations between auditory and visual electrode pools

**FIGURE 4:**
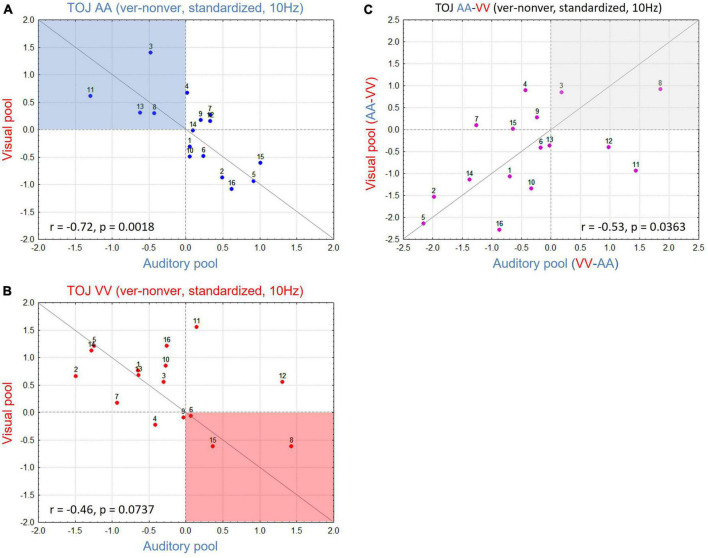
**(A)** Scatterplot for all participants in the auditory (AA) temporal-order judgment (TOJ) condition with V-pool plotted over A-pool for 10 Hz. Red dotted horizontal and vertical lines denote standardized value = 0, gray line is the unity line (45°). Active inhibition hypothesis (AIH) expects data in the blue shaded area with higher activation in V-pool reflecting distractor suppression in the task irrelevant modality. **(B)** As in panel **(A)** but plotting data for the visual (VV) TOJ condition. Here AIH predicts the data in the red shaded area with higher activation in A-pool reflecting distractor suppression in the task irrelevant modality. **(C)** Difference of **(A,B)** (for each pool difference between the irrelevant—relevant modality). This plot indicates the consistency of the effect for each participant, irrespective of condition (see section “3. Results and discussion” for more details). High values accord with AIH predictions, low values oppose AIH predictions. More individuals show a modulation in contrast to AIH in our sample yielding the result on the group-level to be in contrast to AIH predictions. However, the changes across conditions are for both groups of individuals consistent and systematic.

To study whether the interaction pattern found in the group data also holds on the participant level, we plotted standardized values of differences between V-pool and A-pool for each participant, separately for AA TOJs (Figure 4A) and VV TOJs (Figure 4B). In a strong interpretation of the AIH (e.g., Foxe and Snyder, 2011), location of all data points for the AA TOJ would be expected in the upper left quadrant (Figure 4A, highlighted in blue). This quadrant represents all participants with a desynchronization of α-band power in the congruent modality (here auditory) accompanied by an increase over the incongruent modality (visual). If participants show the opposite sign in the difference standardized value (α-power in the veridical minus non-veridical condition) as in Bernasconi et al. (2011) and in the present report, we expect the data points of all participants to be located in the lower right quadrant. As can be seen in Figure 4A, neither is the case: standardized values are broadly scattered across both quadrants, with relatively high standardized values in A-pool accompanied by relatively low standardized values in V-pool. Next, we tested if this scatter across quadrants is systematic. This scatter, with participants showing strong modulation consistent with AIH, no or little modulation, and strong modulation contrary to AIH, explains the weak modulation dynamics found in AA across the A- and V-pool (cf. Figure 3D). However, and this is the important point, the scatter is not random but systematic [spearman rank test: r = -0.72 (*p* < 0.0018)]: when individuals show modulation, activity at central electrodes increases while it simultaneously decreases over parieto-occipital electrodes and vice versa. Since there are correspondingly individuals of both types, the modulation effect is not reflected in the mean (cf. Boenke et al., 2009).

Plotting the VV TOJ data in the same way (Figure 4B, with AIH highlighted in red) we obtained a similar picture. Again, we found a systematic broad scatter across quadrants along the 45° line (r = -0.46, *p* = 0.07). This analysis was also done for non-standardized values where we obtained a positive correlation. Here, in TOJ VV one participant needed to be excluded as an outlier due to very high α-power baseline levels (once more supporting standardization). A positive correlation for absolute values means that e.g., higher α-power over V-pool is consistently accompanied with higher α-power over A-pool and lower α-power over the former with lower α-power over the latter, respectively. However, the slope was not 1, demonstrating different dynamics over V- and A-pool which could only be teased out by standardized values (or the interaction in the ANOVA).

Finally, to test if this relationship of upregulated α-band power in one modality accompanied by downregulation in the other is consistent across conditions and participants, we subtracted for each ROI the congruent from the incongruent condition (e.g., for A-pool, AA TOJ standardized values were subtracted from VV TOJ standardized values and accordingly for V-pool). By this procedure (Figure 4C), we would expect participants with the largest (in each direction of modulation), consistent α-effects to have an extreme value (at each end of the distribution) in the new scatter plot. Participants showing effects consistent with AIH should show larger values (power over congruent neural networks is relatively decreased) and those with effects consistent with the group mean reported here should show smaller values (power over congruent neural networks is relatively increased). If our data reflect a consistent α-power modulation as we have outlined, we expect data from the V-pool to be positively correlated with data from the A-pool. As Figure 4C shows, this is indeed what we found (r = 0.53, *p* = 0.036). In other words, the direction of α-power modulation cannot be generalized over all participants, but if it is known for one condition in a given participant it is for the other condition determined.

## 4. General discussion

Inspired by studies reporting results which are seemingly irreconcilable with the common view of α/β-band modulations, and by our own work addressing a different research question (cf. section “1 Introduction”), we studied cross-modal prestimulus dynamics in purely auditory and visual TOJs. By employing a spatialized design, asking participants on which side a stimulus was first perceived, we were able to present both conditions within one experimental session without a cue or task switch. Thus, we were able (1) to confirm the results by Bernasconi et al. (2011) who found that higher β-band power (20 Hz) in auditory regions was associated with veridical AA TOJs and (2) to extend their study to include the visual modality, which also allowed us to re-test results which have been interpreted to support the AIH hypotheses also in cross-modal contexts assuming active inhibition of the task-irrelevant modality (Foxe et al., 1998; Fu et al., 2001).

These results demonstrate for the first time the involvement of modality-specific neural networks in TOJs (auditory and visual TOJs contrasted within a single experiment). Moreover, we also found cross-modal prestimulus dynamics, with an interaction between electrode site and TOJ condition: In AA TOJ, α-band power (10–15 Hz) was on average higher over central electrodes (A-pool) and was accompanied by a decrease in α-band power over parieto-occipital electrodes (V-pool), together with the inverse pattern for VV TOJs (Figure 3B). This indicates a double dissociation and is seemingly at odds with the strict interpretation of the AIH, stating that an increase in prestimulus α/β-band power indicates an active distracter suppression mechanism (probably based on pulsed inhibition), while a decrease of power indicates greater stimulus processing. This strict view would also assume that, under all conditions, all participants would show higher α-band power with distracter suppression.

Analyses on the participant level, however, revealed a more diverse pattern (irrespective of whether absolute power, GPS, or standardized values across the electrode array were considered) suggesting that these results can be reconciled. Specifically, correlation analysis revealed that even participants who showed modulations of α/β-band power in the opposite direction to the group mean need not be regarded as outliers or “noise,” because whatever the participant’s specific pattern of activation change (up or downregulated) in the auditory or visual modality was, it was (always, if we disregard individuals who generally had a low alpha level) systematically reversed in the other stimulus condition (Figure 4).

It might be argued that our result is facilitated through our standardized measure across electrodes which may have introduced dependencies across channels. However, there are several reasons which make such an explanation unlikely. Note that the reversal of activation over auditory and visual electrodes *within* a condition is not a trivial consequence of studentization. Due to the high number of degrees of freedom in a 62-channel array it is unlikely that any two sets of up and downregulated channels would exactly correspond to the A-pool and V-pool electrodes. Most importantly, the effect was systematic *across* conditions and *across* participants (Figure 4C) and absolute power values yielded the same pattern of interaction as standardized values (with the disadvantage associated with absolute values mentioned in the section “2 Materials and methods” or section “3 Results and discussion”).

There is another argument against the explanation of active distractor suppression as a sufficient explanation for our data. To actively suppress a distracter any system needs to have some information about it, which was through the cue available in previous work but not in ours. In our study, both the side of stimulation and the target modality were unpredictable, hence making an active suppression mechanism based on them implausible. Moreover, why should there be an upregulation in the auditory modality (and not in any other modality as e.g., tactile) when the visual modality is cued? One explanation for such an observed pattern of up and downregulation may be the binary nature of typical tasks (including ours), that allow participants to accumulate knowledge of the task as a whole (and not a specific stimulus alone) with the consequence that maximum processing efficiency could be reached by maximizing the difference between the opposite activation levels. Consequently, bidirectional activation levels as found here and in other studies may reflect expectation or adaptation to a specific task rather than active distracter processing. Employing a cue in a binary task, as many previous studies have done [e.g., reviewed in Foxe and Snyder (2011), Palva and Palva (2011)] might have facilitated such a view of active suppressing. It would be interesting to see what pattern emerges in the prestimulus modulations when more than two modalities are involved. The question would be whether there is always an opposite modulation to the relevant modality in the non-relevant modalities, or whether a specific (average) pattern emerges for each individual. We tend to the latter, but it remains to be answered in future studies.

Bauer et al. (2014) hypothesized that prestimulus α/β-band modulations were related to expectations (see also von Stein et al., 2000; van Ede et al., 2010). These authors reported that the strength of modulation (prestimulus decrease) is associated with the strength of expectations (precision). However, whereas some of our participants fitted this pattern, most did not. The pattern in the latter participants might be better explained by an alternative hypothesis put forward by Engel and Fries (2010) that prestimulus α/β modulation is an index of strategic top-down modulation. They hypothesized that higher coupling reflects the prediction of *status quo* maintenance and is observed frequently in studies using ambiguous stimuli. A stronger coupling of α/β-power modulation maintaining the *status quo* would suit our observed central tendency and may reflect participants’ maintaining their expectations toward a specific modality. However, again the problem is that we find individuals whose data is opposite to the predicted sign of modulation. A way to see our results in this light would be as follows: our data suggest that certain states prior to the onset of the first stimulus increase the likelihood of better judgments of the order of stimulus pairs. Different states may lead to different strategies for processing the task. Apparently, for different individuals, different states (and resulting different strategies) lead to the same effect (i.e., better discrimination performance). The interaction we found in our data is compatible with the view that it is not important to have a particular strategy, but rather to maintain the strategy once it is in place (out of a multiplicity of possible strategies that are fit in the particular context) for as long as it is (deemed) fit. That different strategies can lead to the same (behavioral) effect has now been shown in several studies [e.g., Sanfratello et al. (2014); reviewed in Seghier and Price (2018)].

### 4.1. Task dependency and complexity

We are aware of one other study investigating temporal performance and prestimulus activity. This study investigated the influence of α/β prestimulus modulation on temporal discrimination of tactile synchrony judgments (SJ). In line with the AIH, and contrary to studies employing TOJs, Lange et al. (2012) reported higher prestimulus β-band activity associated with more non-veridical performance (judging more often non-simultaneous stimulation as being simultaneous) and proposed that higher prestimulus power yields less efficient processing of the second stimulus. Although it is not clear to us how prestimulus power could selectively hamper processing of the second stimulus, such a mechanism would agree with Bernasconi et al.’s (2011) observation that higher prestimulus power in their AA TOJ study was associated with veridical judgments [thus reversed sign of activation compared to the study of Lange et al. (2012)]. A reliably processed first stimulus and a relatively degraded second stimulus, especially when the side is known of the second stimulus when the first stimulus is veridically judged, would facilitate veridical responses in TOJ, and the relative shift of power between modalities we found would suggest it is modality specific. However, once more it cannot explain the interindividual differences found in our study.

Evidently a TOJ is a more complex task than an SJ since it not only demands discrimination of events but additionally their identification. They are also more demanding than purely perceptual tasks (e.g., Hanslmayr et al., 2007; Busch et al., 2009) or *cued* discrimination tasks (e.g., Foxe et al., 1998). Thus, when task demand is increased it is likely that neural network interaction is increased and presumably the number of degrees of freedom of possible neural network states is increased too. This increase not only gives room for the possibility of different behavioral and cognitive strategies to emerge but might also yield higher observed variability across trials or participants with identical number of draws making it *de facto* difficult to generalize a specific brain state for all participants for a given situation.

### 4.2. Reflections on specificity and consistency in the face of complexity

Although a number of recent studies suggest an active involvement of α/β-band modulations in cognitive processing, we are still lacking an overarching theory about oscillations and their functional role in general (cf. Engel and Fries, 2010; Iemi et al., 2017; Keitel et al., 2022). While each of these studies can well explain their separate data sets, in terms of the discussed direction of modulation they do not fit together. In the present study we found both directions of α/β-power modulation across participants. Some participants showed more veridical trials when prestimulus power in the specific modality was decreased; others when it was increased. Again, and importantly, this effect was highly systematic indexed by the reversal of modulation within each participant when the other modality was stimulated. In other words, the sign of the effect within one participant was not predictive of the outcome in a specific condition but if the sign in one condition for a specific participant is known the sign in the other condition is determined. It is important to note that this result is not contradicting the hypothesis of pulsed inhibition (one important feature of the AIH model) in the α/β range *per se*. Still, pulsed inhibition might be the functionally relevant mechanism shaping processing in the system. However, it appears that the specific sign of the modulation in terms of the final behavioral outcome loses relative importance as the demands of the task, and thus the complexity of the neural networks involved, increase with (spatialized) TOJs. We will next elicit what theoretical underpinnings might be responsible for this and what this entails.

**FIGURE 5:**
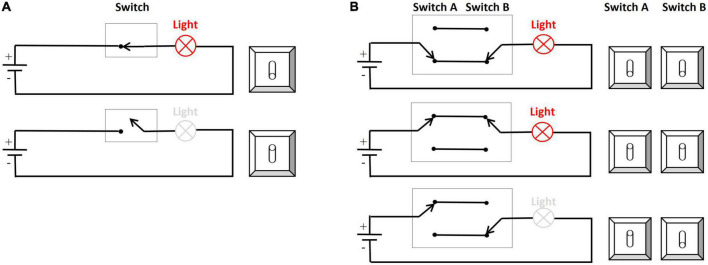
**(A)** A simple system represented by a simple electrical circuit. The switch position (up/down) always predicts the state of the system (closed/open) and the state of the lamp (on/off). **(B)** A more complex electrical circuit. The position of the switch ensemble is no longer informative, because now both switch positions induce the same effect. Instead of the specific switch position, the consistency of the ensemble (switch ensemble) becomes more important. This raises the question to what extent a dichotomous structuring of the world is meaningful for a deeper understanding of complex systems. See text for more details.

Using an analogy, we can illustrate what might underlie the relative loss of specificity as complexity increases. Figure 5A shows a simple electrical circuit whose state can be causally changed by a binary switch (up/down). Provided there is a causal relationship between the outer switch and the inner switch (within the circuit), a specific outer switch position (up/down) is always predictive of the state of the lamp (light on/off; e.g., switch up, circuit closed, light on). The phenomenon we observe with increasing complexity (i.e., when there are multiple ways to close the circuit and thus turn on the light; Figure 5B) is that (1) the light is turned on only when the switch ensemble points consistently in one direction. (2) since now *any* position is associated with “light on” the position of the switch (ensemble) loses its predictive power with respect to the state of the light (on/off, i.e., the switch position is no longer informative about the state of the lamp). The only thing that matters is the consistency and interaction (synergy) of the different options of the system when a certain state is to be realized. Applying this analogy to prestimulus modulation, we can see on the one hand that the reduction of α-power can in principle be accompanied by a higher sensitivity/processing depth (and thus specific in this concrete sense) (in order to influence the state of the system, switching itself remains as a crucial factor). This is (and has been) confirmed especially in relatively simple and well-controlled laboratory experiments. However, in more complex situations, such as in our spatial TOJ task, specificity becomes relatively less important compared to consistency (much like switch position becomes less important in our analogy). This raises the question of how much weight should be given to the question of a specific prestimulus modulation direction in complex contexts. Especially considering the fact that there are individuals who have no clear α-peak (e.g., Grabot et al., 2017).

Overall, this view is plausible and may reconcile many seemingly contradictory interpretations of datasets in the literature, because what is important for coping with changing environments is not so much a fixed and specific direction of modulation that would hold for all participants, all networks, and all situations, but is, independently within each participant, a systematic modulation of an initial activation state that can reliably represent and encode differences between different environments on a sufficient grain of complexity. This type of encoding would be much more resource efficient than having to maintain a specific encoding for every single event.

## 5. Contribution

In general, the literature on the modulation of prestimulus power has tended to focus on group-effects. Despite the growing number of studies, the picture in the literature has become more puzzling than it has led to an overarching theory. The literature now identifies every conceivable way in which the direction of prestimulus α-power modulation can be associated with improved perceptual performance: from reduced prestimulus power, to null effects, to increased prestimulus α-power, all have been associated with improved perceptual performance. Inspired by our work in another research area (Boenke et al., 2009), we examined the robustness of studies that yielded opposite results with respect to the reported group-effect (Foxe et al., 1998; Fu et al., 2001; Bernasconi et al., 2011) and related group-effects to individual-level variability. As shown in our previous study and increasingly in other studies for different levels of biological organization [reviewed in Seghier and Price (2018), Waschke et al. (2021)], the implicit assumption that variability is noise and must be averaged out may prevent deeper insights into (neuro)biological processes. Like us, other research groups are increasingly proposing that variability (i.e., variation) is a fundamental principle in the (biological) world (Montévil et al., 2016) and that variability should be “embraced” as signal rather than condemned as noise (Seghier and Price, 2018). Here, we have shown that this may also be a promising avenue for the prestimulus power literature. Given the increased likelihood of increased variability in a complex task, it is not always clear when to rule out an “outlier” or when to consider this increased variability induced by the outlier as meaningful. However, it is always a wise decision to be guided by theoretical considerations instead of ritually applying models/tests or thresholds.

## Data availability statement

The raw data supporting the conclusions of this article will be made available by the authors, without undue reservation.

## Ethics statement

The studies involving human participants were reviewed and approved by Otto-von-Guericke University. The patients/participants provided their written informed consent to participate in this study.

## Author contributions

LB and FO contributed to conception and design of the study. LB had the idea, performed part of the analysis, did part of the recording, and wrote the first draft of the manuscript. AH performed part of the analysis and contributed to part of the interpretation. DA, FO, and MS drafted parts of the manuscript. All authors revised the manuscript, read it, and approved the submitted version.
